# Nuclear Factor Erythroid 2-Related Factor 2 Activation Might Mitigate Clinical Symptoms in Friedreich’s Ataxia: Clues of an “Out-Brain Origin” of the Disease From a Family Study

**DOI:** 10.3389/fnins.2021.638810

**Published:** 2021-02-23

**Authors:** Sara Petrillo, Massimo Santoro, Piergiorgio La Rosa, Alessia Perna, Maria Giovanna Gallo, Enrico Silvio Bertini, Gabriella Silvestri, Fiorella Piemonte

**Affiliations:** ^1^Unit of Muscular and Neurodegenerative Diseases, Ospedale Pediatrico Bambino Gesù, IRCCS, Rome, Italy; ^2^IRCCS Fondazione Don Carlo Gnocchi ONLUS, Florence, Italy; ^3^Division of Neuroscience, Department of Psychology, Sapienza University of Rome, Rome, Italy; ^4^Department of Neurosciences, Università Cattolica del Sacro Cuore, Rome, Italy; ^5^UOC of Neurology, Area of Neuroscience, Fondazione Policlinico Universitario A. Gemelli IRCCS, Rome, Italy

**Keywords:** Friedreich ataxia, oxidative stress, neurodegenerative disease, Nrf2, glutathione

## Abstract

Friedreich’s ataxia (FRDA) is the most frequent autosomal recessive ataxia in western countries, with a mean age of onset at 10–15 years. Patients manifest progressive cerebellar and sensory ataxia, dysarthria, lower limb pyramidal weakness, and other systemic manifestations. Previously, we described a family displaying two expanded GAA alleles not only in the proband affected by late-onset FRDA but also in the two asymptomatic family members: the mother and the younger sister. Both of them showed a significant reduction of frataxin levels, without any disease manifestation. Here, we analyzed if a protective mechanism might contribute to modulate the phenotype in this family. We particularly focused on the transcription factor nuclear factor erythroid 2-related factor 2 (NRF2), the first line of antioxidant defense in cells, and on the glutathione (GSH) system, an index of reactive oxygen species (ROS) detoxification ability. Our findings show a great reactivity of the GSH system to the frataxin deficiency, particularly in the asymptomatic mother, where the genes of GSH synthesis [glutamate–cysteine ligase (*GCL*)] and GSSG detoxification [GSH S-reductase (*GSR*)] were highly responsive. The *GSR* was activated even in the asymptomatic sister and in the proband, reflecting the need of buffering the GSSG increase. Furthermore, and contrasting the NRF2 expression documented in FRDA tissues, NRF2 was highly activated in the mother and in the younger sister, while it was constitutively low in the proband. This suggests that, also under frataxin depletion, the endogenous stimulation of NRF2 in asymptomatic FRDA subjects may contribute to protect against the progressive oxidative damage, helping to prevent the onset of neurological symptoms and highlighting an “out-brain origin” of the disease.

## Introduction

Friedreich’s ataxia (FRDA, OMIM #229300) is the most frequent autosomal recessive ataxia in western countries, with an estimated prevalence of 1:80,000 among Caucasian populations and a mean age of onset at 10–15 years (Cossée et al., 1999; Koeppen et al., 2009). Symptoms appear between 5 and 15 years of age in FRDA, and the brain atrophy begins early in the disease and plateaus in later stages, indicating that the neurodegenerative profile is an early-onset disease manifestation, with progressive mixed cerebellar and sensory ataxia, cerebellar dysarthria, and lower limb pyramidal weakness. However, other systemic manifestations, including hypertrophic cardiomyopathy, diabetes mellitus, kyphoscoliosis, pes cavus, optic atrophy, and sensory deafness can occur (Cossée et al., 1999; Koeppen et al., 2009; Pallardó et al., 2020). Late-onset (26–39 years) and very-late-onset (over 40 years) FRDA variants can also take place, usually presenting with a milder phenotype and lack of systemic manifestations (Koeppen et al., 2011). Frataxin (FXN) is a ubiquitously expressed protein, and its deficiency results in the decrease of mitochondrial copy number, iron accumulation, deficits of respiratory chain complex activities, and increased sensitivity to oxidative stress, thus affecting many different body districts (Vaubel and Isaya, 2013; Martelli and Puccio, 2014). The brain is the predominantly affected tissue in FRDA, but damage to cardiac myocytes and pancreatic beta-cells has also been evidenced (Delatycki and Corben, 2012; Loría and Díaz-Nido, 2015; Franco et al., 2017; Koeppen et al., 2017). Therefore, rather than a “brain disease,” FRDA can be considered a “systemic disease,” with implications that go beyond the brain itself.

**FIGURE 1:**
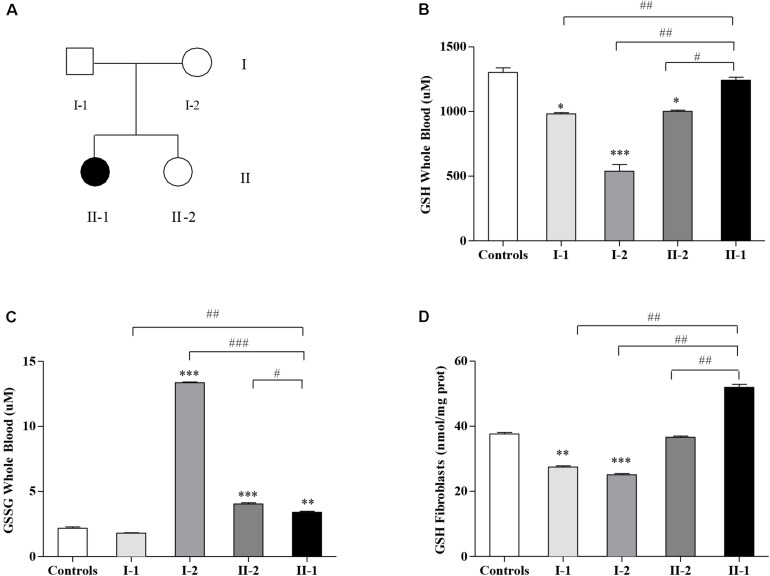
Glutathione homeostasis in Friedreich’s ataxia (FRDA) family’s members. **(A)** Family tree: father (I-1), mother (I-2), younger sister (II-2), and affected proband (II-1) indicated by a black symbol. Reduced glutathione (GSH) **(B)** and oxidized GSSG **(C)** concentrations in the whole blood, and GSH content in fibroblasts **(D)** of I-1, I-2, II-2, and proband II-1 as measured by the enzymatic re-cycling assay. Values are expressed as median ± SEM. Statistical significance was defined as *p < 0.05, **p < 0.01, and ***p < 0.001 with respect to the controls; and ^#^*p* < 0.05, ^##^*p* < 0.01, and ^###^*p* < 0.001 compared with proband II-1.

This study moves from our previous report, where we described a family (Figure 1A) displaying two small expanded GAA alleles not only in the proband (II-1) affected by late-onset FRDA (LOFA) but also in the two asymptomatic family members: the mother (I-2) and the younger sister (II-2) (Santoro et al., 2020). Further studies revealed that both I-2 and II-2 were actually carriers of an expanded GAA allele and of an uncommon (GAAGGA)_66__–__67_ repeat (Santoro et al., 2020), while the father (I-1) was a heterozygous carrier of an expanded allele of about 206 GAA repeats. Although expression studies showed that both the compound heterozygous carriers for the expanded GAA and the (GAAGGA)_66__–__67_ repeat showed a significant reduction of *FXN* mRNA and protein levels in their leukocytes and fibroblasts (Santoro et al., 2020), none of them developed any disease manifestation, supporting that this array represents a benign variant as previously proposed by Ohshima et al. (1999).

To go deeper and understand if a protective mechanism might contribute to modulate the phenotype in this family, here, we report the results of the analysis of redox gene expression profiles in leukocytes and fibroblasts of all family members, particularly focusing on the nuclear factor erythroid 2-related factor 2 (NRF2) and on its glutathione (GSH)-related target genes.

Oxidative stress is a common condition in many neurodegenerative disorders (Barnham et al., 2004), and in FRDA, in particular, it represents one of the most peculiar, although not completely understood, aspects of the pathology (Lupoli et al., 2018). The GAA repeat-mediated FXN depletion leads to mitochondrial iron accumulation in the disease, causing reactive oxygen species (ROS) generation and lipid peroxidation (La Rosa et al., 2020c; Turchi et al., 2020a). As NRF2 regulates many genes directly involved in counteracting oxidative stress and NRF2 signaling axis is defective in FRDA (Paupe et al., 2009; Cuadrado et al., 2019; Petrillo et al., 2019), the evaluation of NRF2 expression in this family can help to open a window on new protective factors potentially buffering the FRDA symptomatology. NRF2 also modulates the cellular levels of GSH, which previously was found impaired in FRDA patients (Piemonte et al., 2001; Pastore et al., 2003) and whose equilibrated ratios between GSH and its oxidized form GSSG are crucial in maintaining the cellular redox balance (Schafer and Buettner, 2001). Thus, we further measured the GSH and GSSG content in family’s members, to evaluate their ROS detoxification ability.

By this study, we ask if a differential expression of NRF2 or a dysregulated GSH homeostasis between symptomatic and asymptomatic family’s members may represent a distinctive tract able to confer the clinical protection.

## Results

### The Glutathione Homeostasis Is Dysregulated in the Family

The GSH content has been measured in blood (Figure 1B) and in fibroblasts (Figure 1D) of FRDA family’s members (Figure 1A). As reported in Figure 1B, the GSH balance was dysregulated in blood, with the GSH levels surprisingly high in the affected proband II-1, approaching the controls’ values (1,242 ± 23 vs. 1,302 ± 37 μM controls), whereas the asymptomatic mother I-2 (539 ± 53 μM) and sister II-2 (1,002 ± 8.2 μM) showed low GSH concentrations, as well as father I-1 (972 ± 0.6 μM). In parallel, the GSSG, which represents the oxidation product of GSH, was low in the proband II-1 (3.4 ± 0.08 μM), with respect to the consistently high GSSG levels found in the blood of the unaffected mother I-2 (13.4 ± 0.06 μM) and to the mild but significant rise in that of the younger sister II-2 (4.04 ± 0.09 μM, vs. 2.18 ± 0.10 controls, Figure 1C). The father (I-1) showed no significant differences with respect to the controls. This trend was confirmed in fibroblasts (Figure 1D), with high GSH levels in II-1 (50 ± 0.88 nmol/mg prot.) and low concentrations in I-2 (25 ± 0.33 nmol/mg prot.), II-2 (36 ± 0.37 nmol/mg prot.), and I-1 (27 ± 0.35 nmol/mg prot.).

### The Glutathione-Related Genes Are Differently Expressed in the Family’s Members

**FIGURE 2:**
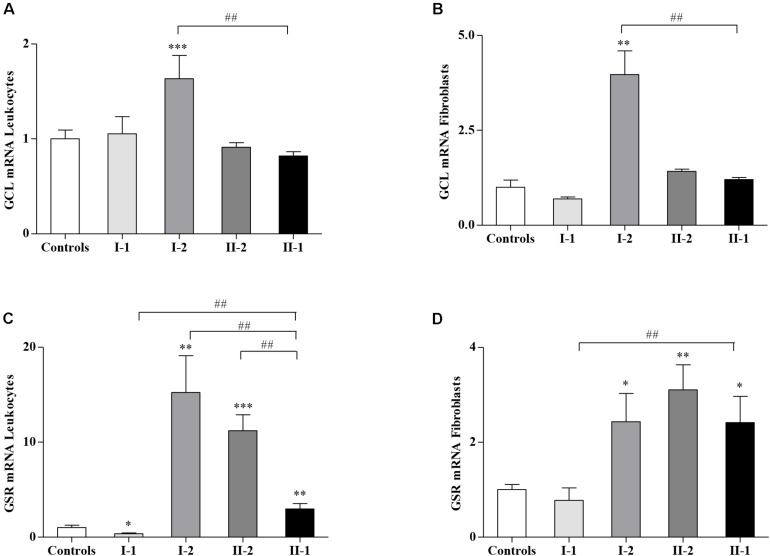
Glutathione-related genes in Friedreich’s ataxia (FRDA) family. The expression of glutamate–cysteine ligase (*GCL*) and glutathione S-reductase (*GSR*) was analyzed by quantitative real-time PCR (qRT-PCR), respectively, in leukocytes **(A,C)** and fibroblasts **(B,D)** of the I-1, I-2, II-2, and proband II-1. Values represent median ± SEM. Statistical significance was defined as *p < 0.05, **p < 0.01, ***p < 0.001 with respect to the controls and ^##^*p* < 0.01 compared with proband II-1.

Given the different amounts of GSH and GSSG in affected and unaffected members of the family, we asked if the GSH-related genes, responsible for the GSH homeostasis in cells, could be dysregulated in the family. Thus, we analyzed the expression of glutamate–cysteine ligase (*GCL*), the gene coding for the step-limiting enzyme of the GSH synthesis, and the GSH S-reductase (*GSR*) gene, implicated in the re-cycling of the GSH from its oxidized form GSSG. As reported in Figure 2, while the GCL expression levels in I-1, II-2, and proband II-1 were comparable with those of the controls (Figures 2A,B), the asymptomatic mother (I-2) showed a significant upregulation of the GCL gene, either in leukocytes (Figure 2A) or in fibroblasts (Figure 2B), probably as a response to the low availability of GSH (Figures 1B,D). The expression of GSR, which reduces the GSSG re-establishing a correct GSH/GSSG ratio, was highly activated in I-2 and II-2, both in leukocytes (Figure 2C) and in fibroblasts (Figure 2D), thus reflecting the need to neutralize the GSSG overload (Figure 1). The GSR gene was activated even in the leukocytes (Figure 2C) and in fibroblasts of the proband II-1 (Figure 2D), who displayed mild but nevertheless significant increase in GSSG concentration (Figure 1). The I-1 showed no significant differences in GCL and GSR expression neither in leukocytes (Figures 2A,C) nor in fibroblasts (Figures 2B,D), with respect to the controls. Overall, these findings demonstrate a strong reactivity to the FXN deficiency of the GSH system, particularly in the I-2, where it was greatly responsive.

### Nuclear Factor Erythroid 2-Related Factor 2 Is Activated in the Asymptomatic Members of the Family (I-2 and II-2)

**FIGURE 3:**
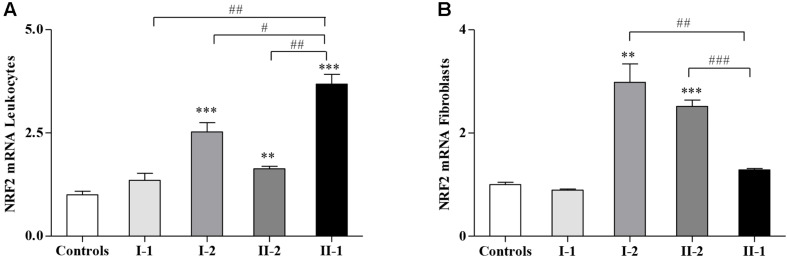
Nuclear factor erythroid 2-related factor 2 (*NRF2*) gene expression in Friedreich’s ataxia (FRDA) family. Quantitative real-time PCR (qRT-PCR) analysis of NRF2 transcripts in leukocytes **(A)** and in fibroblasts **(B)** of I-1, I-2, II-2, and proband II-1. Values represent median ± SEM. Statistical significance was defined as **p < 0.01 and ***p < 0.001 with respect to the controls; and ^#^*p* < 0.05, ^##^*p* < 0.01, and ^###^*p* < 0.001 compared with proband II-1.

Considering that the GSH-related genes are regulated by NRF2, whose expression is impaired in FRDA patients and in preclinical models of FXN deficiency (Paupe et al., 2009; D’Oria et al., 2013; Shan et al., 2013; La Rosa et al., 2019, 2020d; Petrillo et al., 2019; Turchi et al., 2020b), we evaluated if *NRF2* might be differently expressed in the family. Interestingly, as reported in Figure 3, *NRF2* was not induced in the fibroblasts of the proband II-1 (Figure 3B) but highly stimulated in leukocytes (Figure 3A). It is important to note that the symptomatic proband II-1 was under idebenone therapy at the time of blood collection, and idebenone is a well-known NRF2 inducer (Petrillo et al., 2019).

*NRF2* was also significantly activated in leukocytes (Figure 3A) and in fibroblasts (Figure 3B) of I-2 and II-2, while its expression in the I-1 was comparable with that of the controls (Figures 3A,B).

## Materials and Methods

This study was conducted in agreement with the Declaration of Helsinki, and its design fulfilled the guidelines of all involved institutional ethical boards. RNA, and protein samples were extracted from peripheral blood leukocytes or cultured fibroblasts obtained from punch skin biopsies from all family members who gave a written informed consent authorizing storage and use of clinical data and biological samples for diagnostic and clinical research purposes.

### Family Description

The proband (II-1) was a 43-year-old female whose symptoms started at the age of 35, with slowly progressive gait, balance, and mild speech impairment. Her family history was negative (Figure 1A). She first came to our attention at the age of 39 years, and neurological examination documented gaze evoked nystagmus, mild cerebellar dysarthria, gait ataxia, limb in coordination with positive Romberg sign, absent deep tendon reflexes, and bilateral Babinski sign; antibodies, serum alpha-fetoprotein, vitamins B12 and E, and lactic acid levels were all negative. Two pathological GAA expansions of approximately 206 (GAA1) and 473 (GAA2) repeats have been documented in the proband (Santoro et al., 2020).

The asymptomatic 36-year-old sister (II-2) displayed two expanded alleles apparently corresponding to 146 (GAA1) and 176 (GAA2) repeats (Santoro et al., 2020). During 3 years of follow-up, symptoms slowly progressed in II-1, as expected; instead, II-2 did not develop any FRDA manifestation.

Finally, the 73-year-old mother (I-2) carried two GAA expansions of approximately 139 (GAA1) and 389 (GAA2) repeats, though detailed clinical neurological evaluation documented the absence of symptomatology (Santoro et al., 2020).

### Blood Sample Collection

Blood samples from all family members were collected into 5% EDTA Vacutainer tubes (Becton Dickinson, Rutherford, NY) and fractionated as follows: 1 ml was stored at -80°C immediately after drawn for GSH determinations; 1 ml was destined to GSSG measurements and stored at -80°C, until analysis; and 5 ml of whole blood was used for isolation of leukocytes by 10% dextran.

After 45 min at room temperature, the upper phase containing leukocytes was centrifuged at 1,125 × g (5 min) and washed with 0.9% NaCl, until a clear pellet was obtained. Leukocytes have been stored at −20°C until the RNA extraction.

### Cell Cultures

Skin biopsies were taken from all family members and three age-matched controls. Fibroblasts were grown in Dulbecco’s modified Eagle’s medium supplemented with 10% fetal bovine serum, 50 units/ml of penicillin, 50 μg/ml of streptomycin, 0.4% (v/v) amphotericin B (250 μg/ml), and 1 mM of sodium pyruvate at 37°C in 5% CO_2_, as reported in Pastore et al. (2003). Fibroblasts were grown to 70% confluence. The assays were performed in triplicates, and cells were used at similar passage numbers.

### GSH and GSSG Determination

GSH and GSSG levels have been detected using the enzymatic re-cycling assay, as previously reported (Petrillo et al., 2019). Briefly, samples have been de-proteinized with 5% (w/v) sulfosalycilic acid (SSA; Sigma-Aldrich, St. Louis, MO, United States), and the GSH content was determined after dilution of the acid-soluble fraction in Na–phosphate buffer containing EDTA (pH 7.5). To prevent an overestimation of GSSG due to the oxidation of thiols during sample manipulation, blood samples have been collected in tubes prefilled with 30 mM of *N*-ethylmaleimide (NEM) (Giustarini et al., 2013). GSH and GSSG concentrations have been measured with the ThioStar^®^ GSH detection reagent (Arbor Assays, Michigan, United States), using, respectively, GSH and GSSG as standards (Sigma Chemicals, St. Louis, MO, United States). The fluorescence has been measured using an EnSpire^®^ Multimode Plate Reader (Perkin Elmer, Waltham, MA, United States). GSH levels in fibroblasts were expressed as nmol/mg proteins. Protein concentration was determined by the bicinchoninic acid assay (BCA) method (Thermo Fisher Scientific, United States).

### Quantitative Real-Time PCR

Total RNA was extracted from leukocytes and fibroblasts using TRI Reagent (Sigma-Aldrich, St. Louis, MO, United States), according to manufacturer’s protocol. One microgram of each RNA samples was reverse transcribed with the SuperScript^TM^ First-Strand Synthesis system and random hexamers as primers (Life Technologies, Carlsbad, CA, United States). The mRNA of GCL, GSR, and NRF2 was measured by qRT-PCR in an ABI PRISM 7500 Sequence Detection System (Life Technologies, Carlsbad, CA, United States) using Power SYBR Green I dye chemistry. Data were analyzed using the 2^–ΔΔ*Ct*^ method with TATA box binding protein (TBP) as a housekeeping gene and expressed as fold change relative to the controls. Primers used for qRT-PCR are reported in Table 1.

**TABLE 1:** Primers used for qRT-PCR.

Human genes	Sequence 5′>3′
*NRF2*	*Fw*-ACACGGTCCACAGCTCATC
	*Rv*-TGTCAATCAAATCCATGTCCTG
*GCL*	*Fw*-TTGCCTCCTGCTGTGTGATG
	*Rv*-ATCATTGTGAGTCAACAGCTGTATGTC
*GSR*	*Fw*-CACTTGCGTGAATGTTGGATG
	*Rv*-GATTTCTATATGGGACTTGGTG
*TBP*	Fw-CCGAAACGCCGAATATAATCC
	*Rv*-AAATCAGTGCCGTGGTTCGT

### Statistical Analysis

Statistical analysis was performed using the GraphPad/Prism 5.0 Software (San Diego, CA, United States). Statistically significant differences between the controls and family’s members were analyzed using Student’s *t*-test for normally distributed variables. All data are presented as mean ± standard error. Statistical significance was defined as ^∗^*p* < 0.05, ^∗∗^*p* < 0.001, and ^∗∗∗^*p* < 0.001 compared with the controls, and ^#^*p* < 0.05, ^##^*p* < 0.01, and ^###^*p* < 0.001 compared with proband II-1.

## Discussion

This study moves from our previous paper focused on a peculiar family characterized by the presence in two first-degree relatives of the proband, affected by LOFA, of a compound heterozygosity for an expanded (GAA) repeat and a (GAAGGA) repeat at *FXN* locus; both compound heterozygotes are asymptomatic, supporting that the (GAAGGA) repeat would be indeed a benign variant. Yet FRDA studies (Santoro et al., 2020) showed that FXN mRNA and protein levels were markedly reduced not only in tissues of the proband but also in the two asymptomatic compound heterozygotes. This led us to hypothesize that some protective factors may mitigate detrimental effects of FXN deficiency in both subjects; thus, we decided to assess the status of the antioxidant response in that family.

A consequence of the FXN depletion in FRDA is the increase of oxidative stress, and the most credited pathogenic hypothesis is that the FXN-mediated impairment of the mitochondrial iron–sulfur cluster (ISC)-containing enzymes (respiratory chain complexes I–III and aconitase) contributes to the Fenton-mediated overproduction of ROS (Armstrong et al., 2010; Gomes and Santos, 2013; Vaubel and Isaya, 2013; Abeti et al., 2016; Lupoli et al., 2018).

High susceptibility to oxidative stress has been demonstrated in FRDA patients’ fibroblasts in early studies (Wong et al., 1999), and ROS overload was found in yeast (Bulteau et al., 2007; Irazusta et al., 2008), drosophila (Llorens et al., 2007; Anderson et al., 2008; Soriano et al., 2013), and mouse (Al-Mahdawi et al., 2006; Lupoli et al., 2018) disease models. In addition, elevated levels of oxidative stress markers have been found in the blood (Emond et al., 2000; Schulz et al., 2000; Bradley et al., 2004) and cells (Cotticelli et al., 2013; Abeti et al., 2015, 2016, 2018; Petrillo et al., 2019) of FRDA patients.

However, unlike the expected activation of the NRF2-mediated antioxidant defense, the NRF2 signaling pathway is defective in FRDA patients and in preclinical models of FXN deficiency (Paupe et al., 2009; Shan et al., 2013; La Rosa et al., 2020a,b), thus further exacerbating the susceptibility to oxidative stress and its induced defects in the disease (Abeti et al., 2018; La Rosa et al., 2020c,d).

In this family, we analyzed the antioxidant response in all members, particularly focusing on the GSH metabolism and *NRF2* expression, both pathways representing the first antioxidant defense lines in tissues. GSH is the main redox indicator in cells, and previous studies reported decreased levels of this molecule in the blood of FRDA patients.

NRF2 is the principal regulator of the GSH homeostasis by upstream modulating the GSH synthesis (*GCL* gene) and the GSH recycling from its oxidized form GSSG (*GSR* gene). All these actions may actively contribute to counteract the oxidative stress-mediated injury and, potentially, to slow down the onset of symptoms in FRDA.

Our findings demonstrate that the GSH homeostasis was dysregulated in the family (Figure 1), yet with unexpected significantly low GSH concentration in the asymptomatic compound heterozygous I-2 and high levels in the proband II-1. The amount of GSSG was also consistently high in I-2, and a moderate increase was even found in the other compound heterozygous II-2 and in the proband II-1, likely indicating a general activation of the GSH-mediated response. Also, the GSH-related genes were differently expressed in the family members (Figure 2), showing a great reactivity of the GSH system to the FXN deficiency, particularly in the I-2, where the genes of GSH synthesis (*GCL*) and of GSSG detoxification (*GSR*) were highly responsive. The *GSR* gene was activated even in the other compound heterozygous II-2, as well as in the proband II-1, reflecting the need of buffering the increase in GSSG.

However, as the imbalance of GSH levels did not allow explaining the lack of symptoms in the FXN-deficient compound heterozygous I-2 and II-2, we focused our attention on NRF2, the upstream regulator of GSH homeostasis, which is usually depleted under conditions of FXN deficiency.

Contrasting the reduced NRF2 expression documented in FRDA tissues, NRF2 was significantly activated in both leukocytes and fibroblasts of the two asymptomatic compound heterozygous I-2 and II-2 (Figure 3), suggesting that the occurrence of an endogenous stimulation of this transcription factor in these subjects might translate into protective and preventive effects on the symptomatology.

Instead, NRF2 was downregulated in the fibroblasts of the LOFA proband II-1, yet it was activated in her leukocytes (Figure 3), where it might be related to the effects of the idebenone treatment. Indeed, the proband was under idebenone therapy at the time of blood collection, and idebenone is known to activate *NRF2* expression in FRDA patients (Petrillo et al., 2019; La Rosa et al., 2020a).

Thus, in the LOFA proband, NRF2 is, as expected, constitutively low in fibroblasts, whereas it is exogenously activated in leukocytes by idebenone, but in both the asymptomatic compound heterozygous carriers, NRF2 is constitutively upregulated, although both of them would also show decreased FXN expression. So we hypothesize that the occurrence of a widespread upregulation of NRF2 expression in such individuals might contribute to protect the most susceptible tissues against the progressive oxidative damage and the onset of symptoms. Importantly, we suggest that the early administration of NRF2 inducers in patients, particularly in FRDA children, at the first onset of the disease could slow the progression of neurological damage, thus being of great therapeutic help.

**FIGURE 4:**
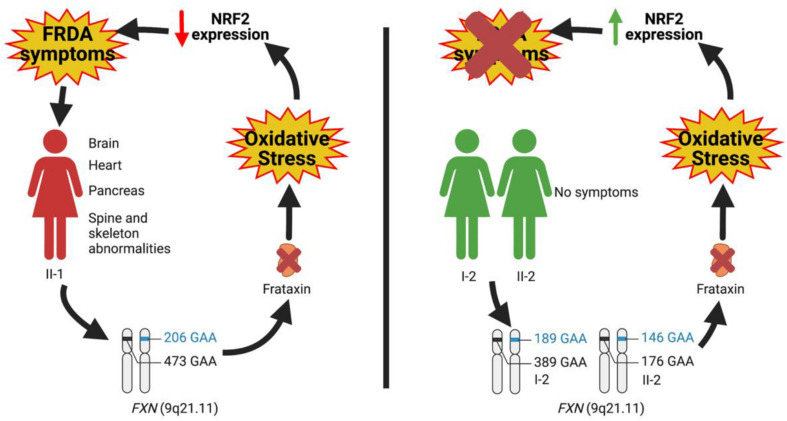
Hypothesis on the role of nuclear factor erythroid 2-related factor 2 (NRF2) as a protective factor antagonizing the insurgence of Friedreich’s ataxia (FRDA) symptomatology.

Overall, by this study, we extend the spectrum of possible effectors responsible for the development of clinical symptoms, thus moving the origin of the disease outside the brain. In this regard, the family we analyzed is paradigmatic since, although all members displayed FXN deficiency, nonetheless some individuals appeared free of symptoms. Such as for Parkinson’s (PD) and Alzheimer’s diseases (AD), also for FRDA, alternative mechanisms, beyond the brain, can be hypothesized to contribute to the pathogenesis of the disease. In particular, our findings support the role of NRF2 as a protective factor whose constitutive upregulation can keep the antioxidant defense above a threshold, able to prevent the appearance of clinical manifestations (Figure 4). Future studies will be needed to expand the panel of NRF2 activities, in order to identify which pathways are more involved in clinical FRDA protection. It is important to note that NRF2 regulates the transcription of approximately 1% of the human genome (Cuadrado et al., 2019) and that beside maintaining the cellular redox homeostasis, multiple cellular processes, including regulation of inflammation, differentiation, proliferation, cell survival, protein homeostasis, and metabolism, are among the functions influenced by its activity (Corenblum et al., 2016; Robledinos-Antón et al., 2017; Cuadrado et al., 2019; Dodson et al., 2019; La Rosa et al., 2019; Turchi et al., 2020b). Two processes were recently shown to be deeply connected to FRDA pathogenesis: (i) ferroptosis, an iron-dependent cell death caused by impaired GSH metabolism, lipid peroxidation, and mitochondrial failure (Cotticelli et al., 2019; La Rosa et al., 2020d; Turchi et al., 2020b); and (ii) inflammation, a mechanism not yet fully understood in FRDA, but potentially involved, as demonstrated in fibroblasts of patients, where the anti-inflammatory heme-oxygenase 1 (HO-1) gene was found to be reduced (Petrillo et al., 2019) and in patients who showed beneficial effects upon treatment with an NF-kB suppressor (Lynch et al., 2019). Although it is undeniable that the NRF2 activation can ameliorate FRDA pathogenesis rescuing, at least in part, the detrimental effects generated by these processes, deeper and more complex regulations could be responsible for the NRF2-mediated protection observed in asymptomatic members of the family. Elucidating these defense mechanisms will be crucial not only in a mitochondrial and systemic disease such as FRDA but also in other oxidative stress-mediated disorders characterized by an out-brain origin (i.e., PD and AD).

## Data Availability Statement

The raw data supporting the conclusions of this article will be made available by the authors, without undue reservation.

## Ethics Statement

The studies involving human participants were reviewed and approved by the Ethics Committee of Bambino Gesù Children’s Hospital (code 1166/2016; date of approval 08/06/2016). The patients/participants provided their written informed consent to participate in this study. Written informed consent was obtained from the individuals for the publication of any potentially identifiable images or data included in this article.

## Author Contributions

SP carried out the experiments and finalized the manuscript. MS, GS, and FP interpreted the data and wrote the manuscript. MG contributed in cell handling. PLR, AP, and EB performed the critical revision of the manuscript for important intellectual content. All authors contributed to the article and approved the submitted version.

## Conflict of Interest

The authors declare that the research was conducted in the absence of any commercial or financial relationships that could be construed as a potential conflict of interest.
